# Electroretinographical Analysis of the Effect of BGP-15 in Eyedrops for Compensating Global Ischemia–Reperfusion in the Eyes of Sprague Dawley Rats

**DOI:** 10.3390/biomedicines12030637

**Published:** 2024-03-13

**Authors:** Barbara Takács, Anna Szilágyi, Dániel Priksz, Mariann Bombicz, Adrienn Mónika Szabó, Beáta Pelles-Taskó, Ágnes Rusznyák, Ádám Haimhoffer, Rudolf Gesztelyi, Zoltán Szilvássy, Béla Juhász, Balázs Varga

**Affiliations:** 1 Department of Pharmacology and Pharmacotherapy, Faculty of Medicine, University of Debrecen, Nagyerdei St. 98, H-4032 Debrecen, Hungary; takacs.barbara@pharm.unideb.hu (B.T.);; 2 Department of Molecular and Nanopharmaceutics, Faculty of Pharmacy, University of Debrecen, Nagyerdei St. 98, H-4032 Debrecen, Hungary; 3 Department of Pharmaceutical Technology, Faculty of Pharmacy, University of Debrecen, Nagyerdei St. 98, H-4032 Debrecen, Hungary

**Keywords:** BGP-15, retina, ischemia–reperfusion, electroretinography (ERG), eyedrops, sulfobutylether-β-cyclodextrin (SBECD), ligation

## Abstract

Retinal vascular diseases and consequential metabolic disturbances in the eye are major concerns for healthcare systems all around the world. BGP-15, a drug candidate small-molecule [O-(3-piperidino-2-hydroxy-1-propyl) nicotinic amidoxime dihydrochloride], has been formerly demonstrated by our workgroup to be retinoprotective both in the short and long term. Based on these results, the present study was performed to investigate the efficacy of BGP in an eyedrop formulation containing sulfobutylether-β-cyclodextrin (SBECD), which is a solubility enhancer as well. Electroretinographical evaluations were carried out and BGP was demonstrated to improve both scotopic and photopic retinal a- and b-waves, shorten their implicit times and restore oscillatory potentials after ischemia–reperfusion. It was also observed to counteract retinal thinning after ischemia–reperfusion in the eyes of Sprague Dawley rats. This small-molecule drug candidate is able to compensate for experimental global eye ischemia–reperfusion injury elicited by ligation of blood vessels in rats. We successfully demonstrated that BGP is able to exert its protective effects on the retina even if administered in the form of eyedrops.

## 1. Introduction

Retinal vascular diseases and consequential metabolic disturbances in the eye are major concerns for healthcare systems all around the world. In several ocular conditions, including age-related macular degeneration, cataract, glaucoma, and advanced stages of diabetic retinopathy [1], ischemia–reperfusion injury is involved. Retinal vascular occlusion—let it be partial or complete—may cause ischemic injury in these diseases, and the following reperfusion further damages the neuronal tissue in the eye, which contributes to impaired vision and visual loss [2]. Oxidative stress presented by ischemia–reperfusion trauma further leads to inflammation, retinal edema, and ultimately necroptosis [3,4]. Retinal vascular abnormalities induce neurodegeneration marked by activation of microglia, increased expression of glial fibrillary acidic protein (GFAP), apoptosis and necrosis of retinal cells, and a substantial decrease in certain layers of the retina [5]. Abnormalities in the retinal tissue lead to retinal dysfunction, which can often be detected prior to additional clinical signs of retinopathies [5,6]. Before the well-established clinical funduscopic analyses were carried out to screen for microangiopathy-related signs such as hemorrhages, hard and soft exudates or cotton wool spots in diabetic retinopathy, for example [7], other less visible changes had already been developed, like thickness reduction [8] and retinal cell apoptosis [9], which result in electroretinographical changes such as smaller a- and b-wave amplitudes, longer implicit times or weaker/missing oscillatory potentials [10], and the same is true of ischemia–reperfusion injury [11,12]. Early detection and management of vascular retinopathy are important for preventing progression and preserving vision as well as starting a proper treatment regime, prior to tissue damage becoming severe enough to significantly impair visual function [13]. Retinal ischemia–reperfusion (I/R) injury is one of the leading causes of visual impairment [14], and effective therapies to prevent or reverse retinal I/R injury have yet to be developed [15].

BGP-15 [O-(3-piperidino-2-hydroxy-1-propyl) nicotinic amidoxime dihydrochloride], a drug candidate small-molecule, was initially developed for its chemoprotective effects against the myelo, nephro-, and neurotoxic effects of cytostatic drugs. Furthermore, BGP-15 (BGP) has been demonstrated to improve type 2 diabetes mellitus-related insulin resistance and entered phase II clinical trials almost a decade ago: it was proven to be safe in short-term toxicological studies in humans [16]. Our workgroup also carried out studies with the agent, in which it was found to be retinoprotective in Goto–Kakizaki rats, a type 2 diabetes mellitus model animal [17]. We also demonstrated long-term efficacy and toxicological evaluation in the Zucker Diabetic Rat, an obese type 2 diabetes model [18]. In these experiments, BGP was able to exert functional preservations, i.e., restoration of decreased electroretinographical a- and b-wave amplitudes [16,17]. Based on these results and bearing drug development aspects in mind, the present study was conducted to evaluate any potential retinoprotective effects of BGP in an eyedrop formulation administered to a rat model of retinal ischemia–reperfusion injury.

## 2. Materials and Methods

### 2.1. Animals

Male adult *Sprague Dawley* (SD) rats (8 weeks of age, 250–270 g, *n* = 20; Janvier Labs., Le Genest-Saint-Isle, France) were housed under standard conditions (22–24 °C) in the animal house of the Department of Pharmacology and Pharmacotherapy, University of Debrecen, Hungary.

The rats were kept under a 12- to 12 h light–dark cycle and had ad libitum access to tap water and standard rat chow. Before the beginning of the experiment, the rats had a 2-week adaptation/acclimatization period.

The animals received humane care, and all experimental procedures were carried out in accordance with the ‘Principles of Laboratory Animal Care’ of the EU Directive 2010/63/EU. All experimental protocols were approved by the local Ethics Committee of the University of Debrecen (8/2020/DEMÁB).

### 2.2. Experimental Protocol

After the 2-week acclimatization period, the rats were randomly assigned into two groups: vehicle-treated and BGP-treated groups (*n* = 10 in each).

At the start of the experiment, ocular ischemia was triggered and maintained for 60 min. After the ischemic period, reperfusion lasted for 1 week, during which the animals were treated with eyedrops. We administered eyedrops with or without BGP twice a day for a week. The eyedrops were dripped into the eye through a pipette tip using a manually adjustable pipette.

In accordance with the 3R rule of animal ethics, to minimize the number of animals needed for the experiment, we inflicted ischemic–reperfusion injury in the left eye of the animals while using the unligated right eyes as a healthy control group. This was also beneficial from the point of view of normalizing the incidental systemic effect of BGP among the treated and untreated groups.

**Figure 1:**
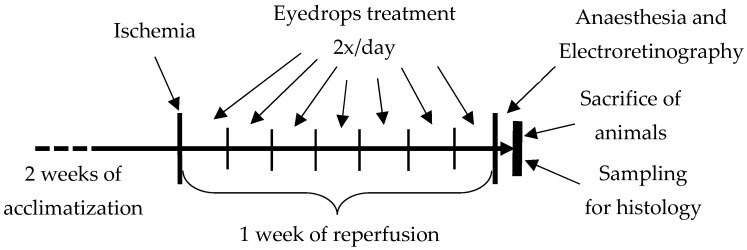
Experimental protocol.

After the 1-week reperfusion and treatment period, animals underwent anesthesia and electroretinographical evaluation took place. The animals were then sacrificed and then eye samples were extracted for histological analysis (Figure 1).

### 2.3. Formulation of Eyedrops

Eyedrops were formulated by our pharmacist colleagues at the Department of Pharmaceutical Technology, University of Debrecen, Hungary. The recipe of the formulation was as follows: 1000 mg BGP (Sigma-Aldrich-Merck KGaA, Darmstadt, Germany) and 1000 mg sulfobutylether-β-cyclodextrin (SBECD) (Cyclolab Ltd., Budapest, Hungary) were dissolved in 8 mL sterile distilled water (Molar Chemicals Ltd., Halásztelek, Hungary), and the pH was set between 7.1 and 7.4 with 1 M NaOH solution (Sigma-Aldrich-Merck KGaA, Darmstadt, Germany). Then, 300 mg hydroxyethylcellulose (Molar Chemicals Ltd., Halásztelek, Hungary) was added to the solution and it was topped up to 10 mL with sterile distilled water. All the ingredients of the formulation have been approved and authorized for pharmaceutical manufacturing of medicines by the European Medicines Agency (EMA) and are included in Pharmacopoeia Europaea inclusive of SBECD [19,20]. The solution was heated to 50 °C and was filtered through a membrane filter with 0.2 µm pores. The concentration of BGP in the above-described eyedrop formulation was 100 mg/mL.

### 2.4. Ocular Ischemia–Reperfusion

The rats were anesthetized with ketamine/xylazine (100/10 mg/kg) (Calypsol, Gedeon Richter Plc., Budapest, Hungary; CP-Xylazin, Produlab Pharma BV, Raamsdonksveer, The Netherlands), then an oxibuprocaine-containing topical ocular anesthetic was administered to the eye (Humacain 4 mg/mL eyedrops, Teva Ltd., Debrecen, Hungary). Thereafter, by ligating the left eyes of the SD rats, experimental ischemia was induced using the previously reported methodology [18]. Concisely, the left eye of each rat was slightly protruded with bent forceps, then a surgical suture composed of polyester fiber (Mersilene, 2 mm, Ethicon Inc., Cincinnati, OH, USA) was inserted behind the eyeball. Then, the suture was slip-knotted around the blood vessels supplying the eye, the optic nerve and the retrobulbar connective tissue. The ligature restricted the blood supply to the retina, which induced ischemia in the left eye. Ischemia was maintained for 60 min and confirmed macroscopically by fundoscopic examination with an ophthalmoscope (Heine mini 2000, HEINE Optotechnik GmbH & Co. KG, Gilching, Germany) and by ocular echography (see below). Then, the occluder was released to allow blood flow via retinal arteries.

### 2.5. Ocular Echography

Ischemia and reperfusion were also confirmed by ultrasound imaging (Vevo 3000, Fujifilm Visualsonics Inc., Toronto, ON, Canada; MX550D transducer at 32 MHz) as detailed before [18]. Briefly, rats were under anesthesia due to the ischemic ligation protocol as mentioned above and were laid on a temperature-controlled pad in a prone position. Using a contact gel (Aquasonic100, Parkerlab Inc., Fairfield, NJ, USA) standard color Doppler was recorded in longitudinal view (9 mm depth, 55°, 0.27 mm gate size). Blood flow of the short ciliary artery seized upon ligation, and was restored after reperfusion, as analyzed by the software of the ultrasound system (VevoLab ver 5.1, Fujifilm Visualsonics Inc., Toronto, ON, Canada).

### 2.6. Electroretinography

A Ganzfeld-type flash electroretinography (ERG) visual monitoring system was used for stimulus generation and data acquisition (Hand-held Multi-species ElectroRetinoGraph (HMsERG), OcuScience, Henderson, NV, USA). ERG measurements were performed according to a previously described method [18]. Vehicle-treated (*n* = 10) and BGP treated (*n* = 10) groups were anesthetized with a mixture of ketamine–xylazine (100/10 mg/kg). After deep anesthesia was reached, mydriasis was induced by topical application of cyclopentolate (Humapent, Teva Ltd., Debrecen, Hungary), and the animals were adapted to the dark for 20 min. Further experimental procedures were performed under dim red light. The animals were positioned on a heated (37 °C) pad (ATC 2000, WPI, Sarasota, FL, USA) in a prone position, and electrodes were placed as follows: a gold-coated corneal contact lens electrode (ERG-jet Contact Lens Electrode, Fabrinal SA, Switzerland) was placed on each eye, while reference and ground stainless steel needle electrodes were inserted subcutaneously above the jaw and tail base, respectively. Conductive gel was applied to the cornea to ensure sufficient electrical contact and to maintain hydration during the entire procedure (Vidisic, Bausch&Lomb, Berlin, Germany). Before measurement, the electroretinography equipment was covered with a Faraday cage. ERGs were recorded from both eyes simultaneously after the animals were placed in the Ganzfeld bowl. The bandpass filter width was 1 to 300 Hz for single-flash recordings that were obtained under both dark-adapted (scotopic) and light-adapted (photopic) conditions. Single white-flash stimulus intensity ranged from −2.5 to 1 log cd·s/m^2^. Light adaptation was performed with a background illumination of 30 cd·s/m^2^ for 10 min before the photopic responses were recorded. For each flash intensity, 10 responses were averaged with an interstimulus interval varying between 2 and 20 s, depending on the flash intensity according to the pre-set protocols of the ERG system. Dark-adapted oscillatory potential (OP) measurements were derived from ERG waveforms recorded to 3000 mcd*s*m^−2^ flash stimuli by filtering with a bandpass of 100 to 300 Hz post-acquisition; the interval between stimulus flashes was 10 s. To measure OP amplitudes, the highest positive peak and lowest negative trough were measured from a baseline set at 0 µV, then the absolute values of the two numbers were added together. The implicit time was the time required to form the highest positive peak after flash stimuli. For each eye, four individual OPs were averaged. Electroretinograms were analyzed with the software supplied by the manufacturer of the ERG system (ERGView 4.380, Ocuscience, Henderson, NV, USA).

### 2.7. Histology

After the extermination of animals, their eye bulbs were immediately enucleated, and the upper part of the eyeball was marked for later positioning. Then, paraformaldehyde solution (PFA, pH 7.4, 4% in phosphate buffer: 10 g paraformaldehyde, 50 µL 10 N NaOH, 25 mL 10× PBS, 200 mL ddH_2_O; Sigma-Aldrich-Merck KGaA, Darmstadt, Germany) was injected into the bulbs, followed by 24 h of immersion to provide appropriate fixation of the retina. On the following day, corneas and eye lenses were removed for a complete removal of the PFA, and the tissue samples were washed in water for 60 min. Then, samples were stored in 70% alcohol until further processing (Sigma-Aldrich-Merck KGaA, Darmstadt, Germany). Dehydration (ascending concentration of ethanol: 70%, 90%, 100%) was the next step, followed by clearing with xylene and embedding into wax (Histowax, Histolab Products AB, Gothenburg, Sweden). Ultimately, sections of 4 µm thickness were cut from the paraffinized eye tissue blocks with a microtome. Sections localized near the optic disk were further processed. After deparaffinization and rehydration of the sections, they were stained with hematoxylin–eosin (H&E) as follows: first, they underwent 10 min incubation in hematoxylin (Gill-type, GHS2128, Sigma-Aldrich-Merck KGaA, Darmstadt, Germany). Then, they were rinsed in running tap water for 10 min until sections turned blue, followed by staining with eosin for 5 min. Images were taken near the optic disk, from the inferior part of the retina with a Nikon Eclipse 80i microscope (Nikon Instruments Inc., Melville, NY, USA) through a 40× objective (Nikon Plan Fluor 40×/0.75 DIC M/N2 ∞/0.17 WD 0.66) with a DS-Fi3 Microscope Camera attached. Measurements were taken with the software of the microscope, Nikon NIS-Elements BR (Ver5.41.00).

### 2.8. Statistical Analysis

GraphPad Prism software (version 8.0, GraphPad Software Inc., La Jolla, CA, USA) was used for statistical analyses. After determining Gaussian distribution of data points using Shapiro–Wilk normality test, multiple comparison tests with post-test were applied: either one-way analysis of variance (ANOVA), if the data passed the normality test, or a non-parametric Kruskal–Wallis test. The result data comparisons were regarded as significant if the probability value turned out to be lower than 0.05. Asterisks were used to indicate significance levels, with 1 to 4 stars (* to ****) in cases of *p* < 0.05, *p* < 0.01, *p* < 0.001, and *p* < 0.0001, respectively. The data in columnal graphs are shown as the mean ± standard error of the mean (SEM).

## 3. Results

**Figure 2:**
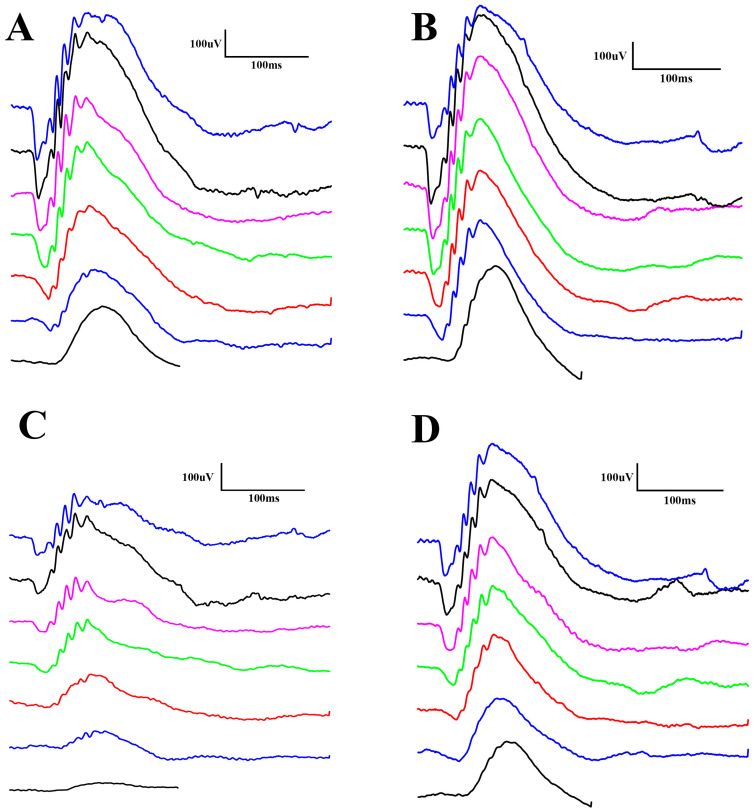
Representative scotopic electroretinographical waveforms elicited by flashlight intensity series (from the bottom (black line) to the top (blue line): 10, 100, 300, 1000, 3000, 10,000, 25,000 mcd*s*m^−2^). (**A**): control no-IR; (**B**): BGP no-IR; (**C**): control IR; (**D**): BGP IR.

**Figure 3:**
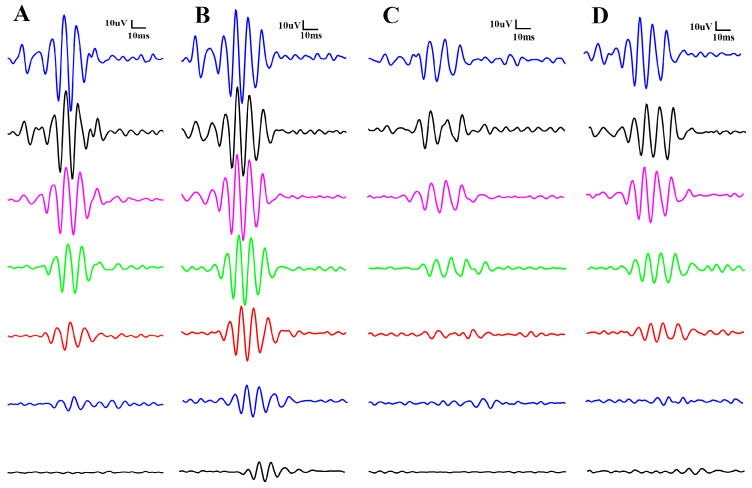
Representative scotopic oscillatory potentials elicited by flashlight intensity series (from the bottom (black line) to the top (blue line): 10, 100, 300, 1000, 3000, 10,000, 25,000 mcd*s*m^−2^). (**A**): control no-IR; (**B**): BGP no-IR; (**C**): control IR; (**D**): BGP IR.

The results of the scotopic electroretinography are shown in Figure 2 as recorded waveforms, while oscillatory potentials (OPs) are shown in Figure 3: A, control no-IR; B, BGP no-IR; C, control IR; D, BGP IR, in both figures. The different colors depict different light stimulus intensities: from the bottom (black line) to the top (blue line): 10, 100, 300, 1000, 3000, 10,000 and 25,000 mcd*s*m^−2^.

The waves in the BGP-treated IR eye-group are higher compared to control IR (Figure 2D vs. Figure 2C) and more closely resemble the physiological form (Figure 2A).

Furthermore, OPs are more pronounced and orderly in the BGP-treated groups compared to the control groups (Figure 3B,D vs. Figure 3A,C, respectively).

**Figure 4:**
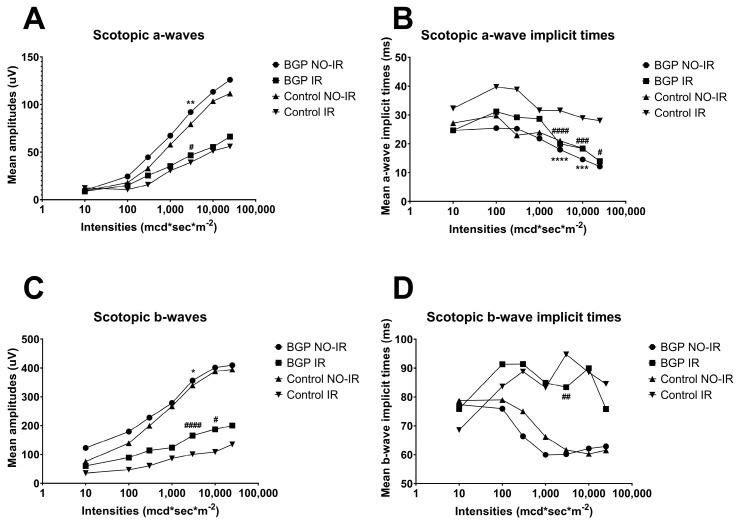
Results of scotopic ERG measurements plotted against increasing flashlight intensities (mcd*s*m^−2^). (**A**): Scotopic a-wave mean amplitudes (µV); (**B**): scotopic a-wave mean implicit times (ms); (**C**): scotopic b-wave mean amplitudes (µV); (**D**): scotopic b-wave mean implicit times (ms). Dots represent BGP-treated group NO-IR eyes, squares BGP-treated IR eyes, upward-pointing triangles represent control group NO-IR eyes and downward-pointing triangles represent control group IR eyes. All values are presented as group means. Statistically significant comparisons are marked with * in case of BGP NO-IR vs. control NO-IR comparisons, and # in case of BGP IR vs. control IR comparisons. The number of markers represents the statistical significance of the comparison * or # *p* < 0.05; ** or ## *p* < 0.01; *** or ### *p* < 0.001; **** or #### *p* < 0.0001.

**Figure 5:**
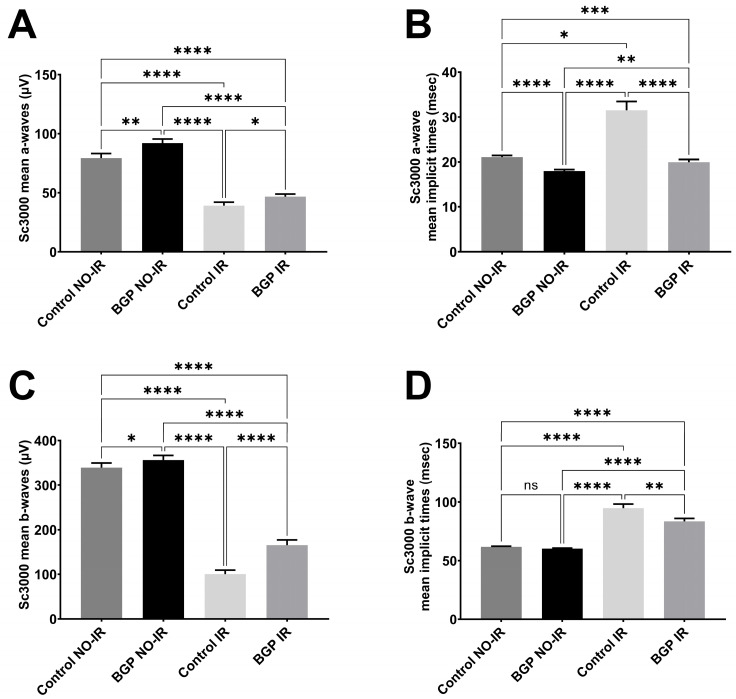
Statistically most important comparisons in scotopic ERG measurements; flashlight intensity: 3000 mcd*s*m^−2^. (**A**): Mean a-wave amplitudes of the different groups (µV); (**B**): mean a-wave implicit times (ms); (**C**): mean b-wave amplitudes (µV); (**D**): mean b-wave implicit times (ms). All results are plotted as group mean ± SEM. ns = no significant difference. Statistically significant comparisons are marked with * *p* < 0.05; ** *p* < 0.01; *** *p* < 0.001; **** *p* < 0.0001.

**Figure 6:**
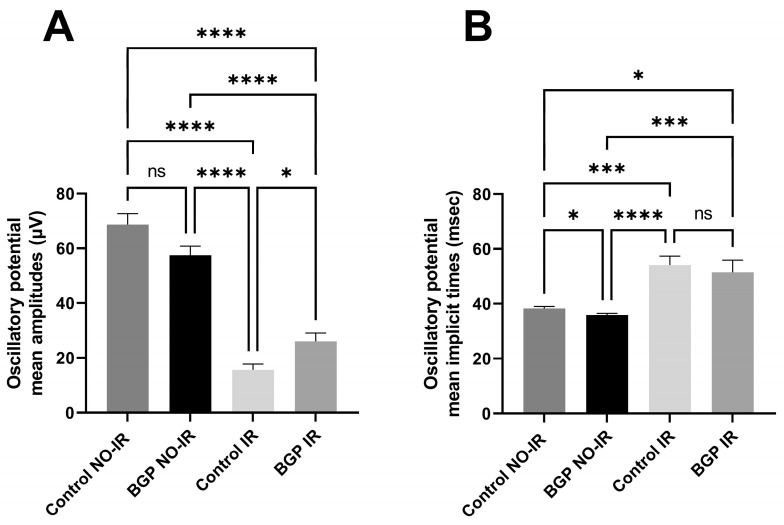
Results and comparisons of dark-adapted oscillatory potential amplitudes and implicit times in different groups, flashlight intensity: 3000 mcd*s*m^−2^. (**A**): Mean oscillatory potential amplitudes for different groups (µV); (**B**): mean oscillatory potential implicit times (ms). All results are plotted as group mean ± SEM. ns = no significant difference. Statistically significant comparisons are marked with * *p* < 0.05; *** *p* < 0.001; **** *p* < 0.0001.

Measurement results related to scotopic ERG are shown in Figure 4 (dots represent the BGP-treated group NO-IR eyes, squares represent BGP-treated IR eyes, upward-pointing triangles represent the control group NO-IR eyes and downward-pointing triangles represent the control group IR eyes), while the most statistically important comparisons are highlighted in Figure 5 in the following group-order: control NO-IR, BGP NO-IR, control IR, and BGP IR. Measurements related to the oscillatory potentials are shown in Figure 6.

It can be observed that for both scotopic a- and b-waves, BGP-treatment was able to elicit higher mean amplitudes as compared to control group values (Figure 4A,C). For the a-wave implicit time (Figure 4B), the control IR values turned out to be the highest, the IR values of BGP-treated groups were observed to be near the NO-IR control values, although at some intensities they were even shorter; and at most intensities, the shortest a-wave implicit times were provided by the BGP-treated NO-IR group. Similar trends were observed in case of b-wave implicit times, but here, values of IR and NO-IR groups were more separated: implicit times of b-waves seemed to be much more sensitive to ischemia–reperfusion injury, which was alleviated by BGP-treatment at some intensities.

According to Figure 5, at 3000 mcd*s*m^−2^ light intensity, the BGP-treated groups had significantly higher a- and b-wave mean amplitudes (92.09 ± 3.470 and 46.69 ± 2.269 µV for BGP NO-IR and IR group mean a-waves ± SEM, respectively, and 356.3 ± 10.00 and 165.3 ± 11.47 µV for BGP NO-IR and IR group mean b-waves ± SEM, respectively) compared to similar values of the control groups (79.27 ± 3.885 and 39.03 ± 2.884 µV for control NO-IR and IR group mean a-waves ± SEM, respectively, and 339.2 ± 10.40 and 100.3 ± 8.976 µV for control NO-IR and IR group mean b-waves ± SEM, respectively; for a-waves, *p* < 0.01 for BGP NO-IR vs. control NO-IR and *p* < 0.05 for BGP IR vs. control IR comparisons; for b-waves, *p* < 0.05 for BGP NO-IR vs. control NO-IR and *p* < 0.0001 for BGP IR vs. control IR comparisons).

There were significant differences in a-wave implicit time values as well: BGP values (17.98 ± 0.3497 and 19.96 ± 0.5929 ms for NO-IR and IR groups, respectively) were significantly shorter than control values (21.06 ± 0.3748 and 31.53 ± 1.931 ms for NO-IR and IR control groups, respectively; *p* < 0.0001 for both BGP NO-IR vs. control NO-IR and BGP IR vs. control IR comparisons). Similarly, in case of b-waves, a significant difference was measured between BGP IR and control IR values (83.38 ± 2.518 vs. 94.72 ± 3.504 ms for BGP IR vs. control IR, respectively, *p* < 0.01). There was no significant difference between BGP NO-IR and control NO-IR b-wave implicit times (60.11 ± 0.4902 vs. 61.63 ± 0.5596 ms for BGP NO-IR and control NO-IR, respectively).

According to measurements related to oscillatory potentials (Figure 6), both the control NO-IR and BGP NO-IR groups had the highest OP amplitudes, while in the BGP-treated IR group, the treatment elicited higher values compared to the control IR group. There were no significant differences in OP amplitudes between the control NO-IR and BGP NO-IR groups (68.69 ± 3.917 vs. 57.49 ± 2.064 for control NO-IR vs. BGP NO-IR groups). There were statistically significant differences between the control IR and BGP IR groups (15.64 ± 2.064 vs. 26.06 ± 2.977 for control IR vs. BGP IR groups). Statistical analysis of OP implicit times revealed no difference between control IR and BGP IR groups (54.14 ± 3.236 vs. 51.42 ± 4.456 for the control IR vs. BGP IR groups), while between control NO-IR and BGP NO-IR groups, there was a significant difference (38.29 ± 0.7049 vs. 35.93 ± 0.6051 for the control NO-IR vs. BGP NO-IR groups).

**Figure 7:**
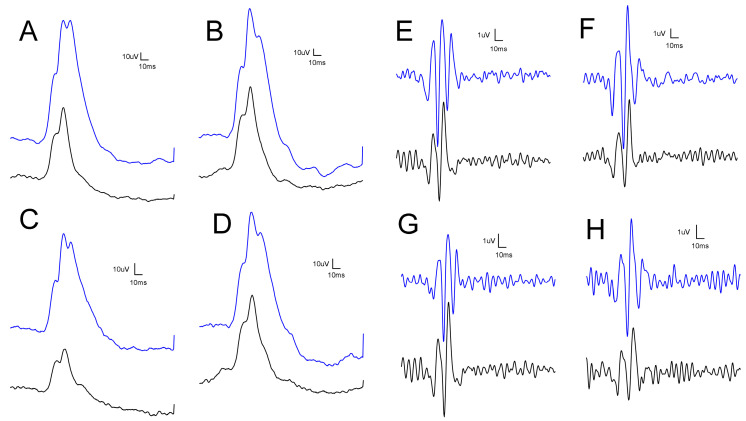
Representative photopic electroretinographical waveforms (**A**–**D**) and photopic oscillatory potentials (**E**–**H**) elicited by light-adapted flashlight intensities (black line for 3000 and blue line for 10,000 mcd*s*m^−2^). (**A**,**E**): Control NO-IR; (**B**,**F**): BGP NO-IR; (**C**,**G**): control IR; (**D**,**H**): BGP IR.

**Figure 8:**
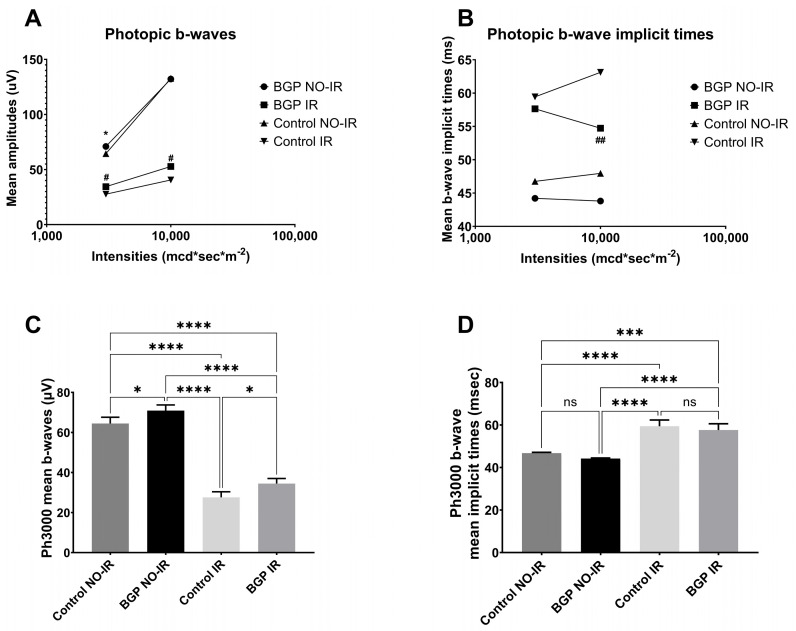
(**A**,**B**): Results of photopic ERG measurements plotted against light-adapted flashlight intensities. (**A**): Photopic b-wave mean amplitudes (µV); (**B**): photopic b-wave mean implicit times (ms). Dots represent BGP-treated group NO-IR eyes, squares represent BGP-treated IR eyes, upward-pointing triangles represent control group NO-IR eyes and downward-pointing triangles represent control group IR eyes. All values are presented as group means. Statistically significant comparisons are marked with * in case of BGP NO-IR vs. control NO-IR comparisons, and # in case of BGP IR vs. control IR comparisons. The number of markers represents the statistical significance of the comparison * or # *p* < 0.05; ## *p* < 0.01. (**C**,**D**): Statistically most important comparisons in photopic ERG measurements; flashlight intensity: 3000 mcd*s*m^−2^. (**C**): mean b-wave amplitudes for the different groups (µV); (**D**): mean b-wave implicit times (ms). All results are plotted as group mean ± SEM. ns = no significant difference. Statistically significant comparisons are marked with * *p* < 0.05; *** *p* < 0.001; **** *p* < 0.0001.

Figure 7 shows waveforms and OPs for light-adapted, photopic electroretinography, whilst Figure 8 illustrates the most important statistical comparisons and measurement results related to photopic ERG.

Photopic ERG measurement was carried out after 10 min background light-adaptation, representative waveforms of which are seen in Figure 7: as demonstrated, BGP treatment was able to restore the physiological course of the curve (compare Figure 7D with Figure 7A), while untreated IR groups showed a more severe deterioration of the waveform (Figure 7C). Similarly, the oscillatory potentials of the BGP-treated IR group (Figure 7H) are more pronounced and orderly compared to control IR (Figure 7G, compared with control NO-IR Figure 7E).

According to the ERG measurements, photopic b-wave mean amplitudes of BGP-treated groups turned out to be higher in almost every comparison (Figure 8A), while implicit times were shorter compared to untreated control groups (Figure 8B). Results of stimulation with photopic light intensity of 3000 mcd*s*m^−2^ are highlighted in Figure 8C,D. There were significant differences in mean b-wave amplitudes between BGP NO-IR and control NO-IR as well as between the BGP IR and control IR groups, *p* < 0.05 in both comparisons (70.95 ± 2.786 vs. 64.46 ± 3.134 µV for BGP NO-IR vs. control NO-IR, and 34.49 ± 2.524 vs. 27.61 ± 2.783 µV for BGP IR vs. control IR groups, respectively). The differences between the mean photopic b-wave implicit times of BGP-treated and control groups did not reach the level of statistical significance.

**Figure 9:**
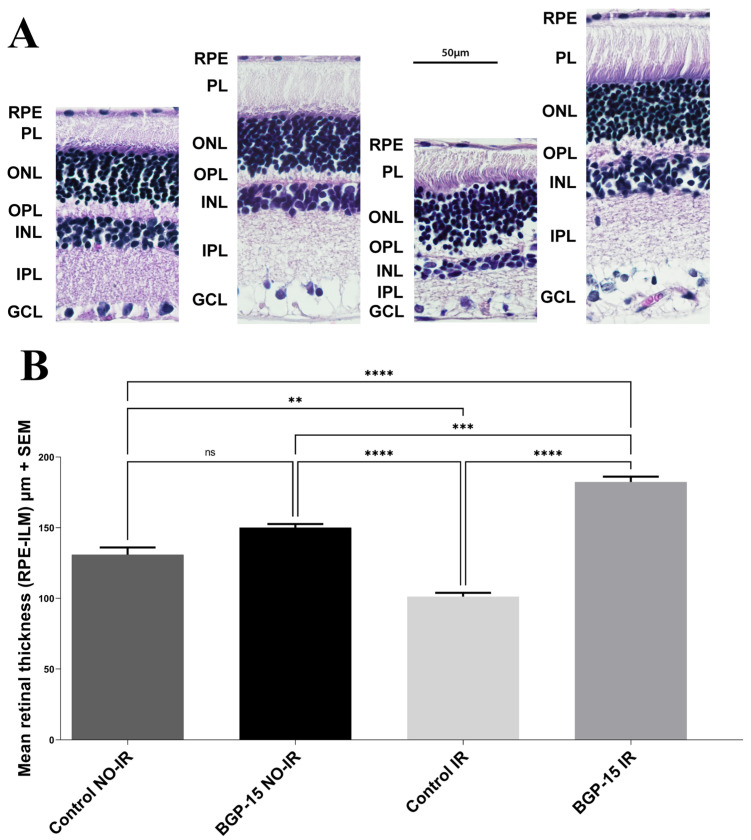
Histology results. (**A**): Representative histological sections of the different groups (from left to right): control NO-IR, BGP NO-IR, control IR and BGP IR. RPE: retinal pigment epithelium; PL: photoreceptor layer; ONL: outer nuclear layer; OPL: outer plexiform layer; INL: inner nuclear layer; IPL: Inner Plexiform Layer; and GCL: ganglion cell layer. (**B**): Graphs showing statistical analysis results of histology sections of the different groups. Data are shown as group mean ± SEM. ns = no significant difference, ** = *p* < 0.01, *** = *p* < 0.001, **** = *p*< 0.0001.

**Figure 10:**
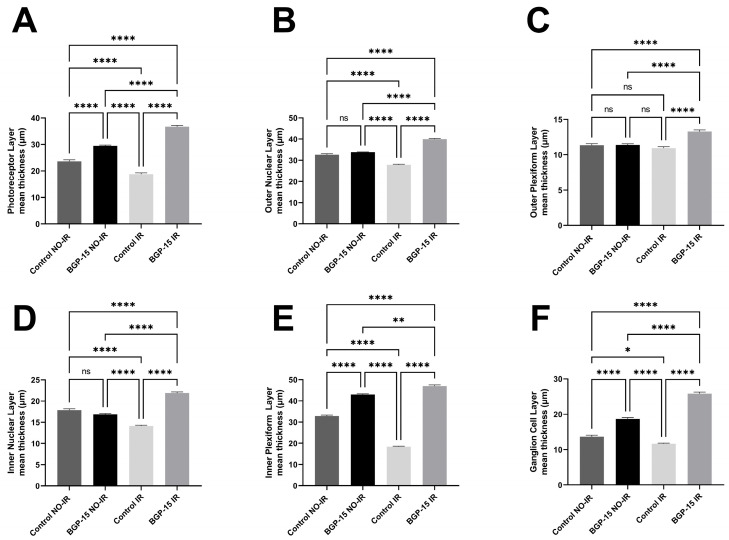
Thickness of the different retinal layers on histological sections. (**A**): photoreceptor layer (PL); (**B**): outer nuclear layer (ONL); (**C**): outer plexiform layer (OPL); (**D**): inner nuclear layer (INL); (**E**): inner plexiform layer (IPL); (**F**): ganglion cell layer (GCL). Data are shown as group mean ± SEM. ns = no significant difference, * = *p* < 0.05, ** = *p* < 0.01, **** = *p*< 0.0001.

The results of the histological analysis are shown in Figure 9 and Figure 10. According to our measurements, the BGP IR group had significantly thicker retina than the control IR group (182.4 ± 3.760 vs. 101.2 ± 2.640 µm, for BGP IR vs. control IR, respectively). The retinal thickness was significantly smaller in the control IR group compared to any other group, while it was the largest in the BGP IR group. There were no significant differences between the BGP NO-IR and control NO-IR groups (150.1 ± 2.553 vs. 130.9 ± 5.118 µm for the BGP NO-IR and control NO-IR groups, respectively).

As shown in Figure 10, all retinal layers except the outer plexiform layer were significantly smaller in the control IR group compared to any other group (18.78 ± 0.5175, 27.93 ± 0.3172, 14.15 ± 0.2031, 18.39 ± 0.2462 and 11.63 ± 0.1921, for photoreceptor layer (PL), outer nuclear layer (ONL), inner nuclear layer (INL), inner plexiform layer (IPL) and ganglion cell layer (GCL), respectively for the control IR group). The BGP-treated IR group, on the other hand, exhibited significantly greater thickness values in every comparison (36.67 ± 0.4810, 39.92 ± 0.3564, 13.30 ± 0.2224, 21.90 ± 0.2496, 47.05 ± 0.5477 and 25.85 ± 0.4335, for PL, ONL, OPL, INL, IPL and GCL, respectively, for the BGP IR group).

## 4. Discussion

Several ophthalmological diseases are related to ischemia–reperfusion injury of the eye, even those that are consequences of metabolic disturbances, e.g., diabetic retinopathy [21]. It is important to manage vascular retinopathies to prevent their progression and preserve vision, and thus new, effective and evidence-based therapies must be developed. Formerly, our workgroup carried out experiments with a small-molecule drug candidate, BGP, O-(3-piperidino-2-hydroxy-1-propyl) nicotinic amidoxime dihydrochloride. We found BGP to be protective in diabetic retinopathy in both the short and long term [16,17]. Based on these results, the present study was performed to investigate the efficacy of BGP in an eyedrop formulation, since topical administration is a frequently used method when treating ophthalmic diseases. Physiologically, the retinal endothelial cells and retinal pigmented epithelium provide the inner and outer blood–retinal barrier, which prevents paracellular movement of hydrophilic compounds. For the drug to reach the posterior segment, it must first be absorbed through the corneal and conjunctival pathways to achieve therapeutical concentrations in the retina [22]. Less than 3% of the drug passes through the cornea [23]; however, certain tissues can bind drugs, increasing the concentration of the drug in the eye: e.g., a considerable binding of topically applied beta-blockers to the retinal tissues has been described previously [24]. In co-operation with the pharmaceutical technology department of our university, in the present study, we assessed the effect of an eyedrop containing BGP and sulfobutylether-β-cyclodextrin (SBECD), a complex-forming, solubility-enhancing cyclic oligosaccharide with a donut-shaped ring structure [25]. Given that BGP shares a structural resemblance with propranolol [26], we proposed that by enhancing the bioavailability of BGP, this formulation may be able to deliver the retinoprotective effects of the agent to the site of action even in the form of an eyedrop.

To distinguish the impact of BGP from cyclodextrin, animals in the control group were treated with vehicle, meaning they were given eyedrops without BGP. Therefore, any observed differences between the control and treated groups can be attributed to BGP—this is an indirect argumentation. A limitation of the study, however, is that the concentration of BGP was measured neither from the blood nor the aqueous humor, and thus, we cannot directly prove whether BGP successfully entered the aqueous humor, or if the measured effects resulted from systemic BGP ingested after it drained through Schlemm’s canal. Future experiments are planned by our workgroup to determine the answer to this. Nevertheless, regardless of the route of entry, the present study registered significant retinoprotective effects of BGP after administration in an eyedrop formulation.

Ischemia–reperfusion injury is known from the scientific literature to distort ERG waveforms and deteriorate a- and b-waves [27,28]. Similarly, in our experiment, these waves were decreased both in scotopic (Figure 2) and in photopic electroretinograms (Figure 7A–D), but treatment with BGP attenuated this change (Figure 4, Figure 5 and Figure 8), which is a novel result. This might be expected, as BGP was already known to be protective against ischemia–reperfusion injury in the heart [29,30] and was proven to be efficient against diabetic retinopathy [16,17]. However, this is the first demonstration which shows that BGP is able to improve a- and b-waves that have deteriorated due to ischemia–reperfusion injury. It is not uncommon to see the retinoprotective effects of an agent that is able to induce heat shock proteins (HSPs), as in the case of heme-oxygenase 1 inducer sour cherry seed extract [31] and alpha-melanocyte-stimulating hormone [32] or HSP co-inducer bimoclomol [33]. BGP protects against heat-, metabolic- and oxidative stress situations through different pathways, including (HSP) induction [34], lipid-raft modification [35] and changing expression of proteins [16,17]. BGP, accumulated in mitochondria, was found to be protective against apoptosis and necrosis in hydrogen peroxide-induced cell death [36] and was able to prevent neuronal death in a mouse model of impaired mitochondrial function [37].

Electroretinographical oscillatory potentials are known to be attenuated in case of diabetes mellitus—even before microvascular complications become visible during funduscopy [38,39]—or similarly in case of ischemia–reperfusion injury [40,41], as in our study as well (Figure 3 and Figure 7E–H). According to the scientific literature, anti-ischemic treatments may restore OPs that have deteriorated due to ischemia–reperfusion damage [42,43], but the present article is the first to describe the OP-restoring effect of BGP (Figure 6).

Ischemia–reperfusion injury and, similarly, oxidative stress (e.g., by H_2_O_2_) are known to increase the latency times (i.e., implicit times) of a- and b-waves in the electroretinogram [44,45,46]. This was evident in our electroretinographical measurements as well. Nevertheless, the present study is the first to demonstrate the implicit time-decreasing effect of BGP (Figure 4B,D and Figure 8B). Similar anti-ischemic retinal protection of other agents was shown by various authors [43,47], although some even reported unchanged implicit times despite other evident effects on the electroretinogram [48].

According to our histology results, BGP was able to preserve retinal thickness, counterbalancing the retina-thinning effect of ischemia–reperfusion, which is a novel result (Figure 9 and Figure 10). A similar protective effect is a hallmark of other anti-ischemic agents as well [31,32,49]. It is well documented that a common consequence of ischemia–reperfusion injury is edema [46,50], and then necro-apopto-autophagy, i.e., initiation of different cell-death mechanisms, resulting in thinning of retinal layers [47,51,52]. Edema, however, may be transient, which implies a reversible injury as well [46,53]. This was the case in our study, since the electroretinograms of BGP-treated animals showed significantly better retinal function than those of untreated animals, and thus the retinal cells remained viable. Similarly, in clinical scenarios, the ERG recordings, which correlate with retinal function even when edema is present, can be utilized to assess both the functionality of the retina and its response to therapy [54]. Thus, the retinal layer thickness differences observed between treated and untreated NO-IR groups can be considered negligible. It is plausible that in the treated IR group, BGP would have exerted its retinal thickness-preserving effect over a longer period of time than our experiment lasted. Although we did not continue our study to measure retinal thickness and ERG at later time-points—a limitation of the study—based on a former study conducted by our workgroup [17], we already know that BGP is functionally effective even if administered for a long time. This further corroborates that the increase in retinal thickness seen on histology sections in the present study might be a late and most probably counteracted edema.

Nevertheless, we have to take into account that the differences seen between treated and untreated NO-IR groups in any measurement throughout the study may be altered by the ligation the animal suffered on its other eye as compared to a healthy animal model. According to the fellow eye phenomenon discussed elsewhere, changes may develop in the unaffected eye following ischemia of the other eye [55]. Nonetheless, by involving a new, healthy animal individual, we would have introduced an unavoidable error factor, namely the individuality (any individual differences) between animals. Furthermore, based on translational aspects of our animal study, it is advisable to use an internal control, because in most clinical cases, there is a fellow eye next to the affected eye, and thus we can see the effect of the treatment on the fellow eye. The relevancy of the comparison to a healthy eye would be to see the effect of the treatment as a prevention, but this was not among the purposes of the current study. Furthermore, according to the rules of 3R for laboratory animal studies (replacement, reduction, refinement), involving the minimal necessary and number of animals into a study must be a priority of any researchers carrying out experiments on laboratory animals.

The directions of future research involve investigating further molecular morphological and biological targets in the action mechanism of BGP. Our workgroup already identified several effector molecules, the levels of which are changed in the eyes of ischemia–reperfusion-related diabetic animal models in response to BGP treatment, including sirtuin 1 (SIRT1), matrix metalloproteinase 9 (MMP9), heat shock protein 70 (HSP70) and nuclear factor kappa B (NFkB) [16,17]. Furthermore, future perspectives include research aimed toward understanding the absorption of BGP from the eyedrop formulation or the mechanism of how it reaches the retina; however, for this purpose, we first have to develop new techniques for measuring BGP concentration from tissues samples.

In summary, we successfully demonstrated that BGP is able to exert its protective effects on the retina even if administered in the form of eyedrops, and validating this was our primary goal. In this study, BGP was shown to improve retinal a- and b-waves, shorten their implicit times and restore oscillatory potentials after ischemia–reperfusion. It was also observed to counteract retinal thinning in IR eyes of Sprague Dawley rats. This small-molecule drug candidate is able to compensate for experimental global eye ischemia–reperfusion injury in rats elicited by ligation of blood vessels.

## Data Availability

Datasets are available on request from the authors; however, restrictions may apply, as the datasets presented in this article are the property of the University of Debrecen. Requests to access the datasets should be directed to the authors.
